# Wild-Type Transthyretin Amyloidosis: A Prevalent and Underdiagnosed Cause of Heart Failure With Preserved Ejection Fraction

**DOI:** 10.7759/cureus.62623

**Published:** 2024-06-18

**Authors:** Bruno Stehlik, Konstantinos Sideris, Lina Brinker, Jill Waldron, Spencer Carter

**Affiliations:** 1 Department of Cardiovascular Medicine, University of Utah, Salt Lake City, USA

**Keywords:** wild-type attr amyloidosis, hereditary attr amyloidosis, heart failure cardiac amyloidosis, heart failure with preserved ejection fraction, transthyretin amyloid, transthyretin amyloid cardiomyopathy

## Abstract

Cardiac amyloidosis is an infiltrative disease characterized by the extracellular deposition of misfolded protein in the myocardium, leading to increased stiffness and an eventual restrictive cardiomyopathy. The slow onset of symptoms and overlap with other cardiomyopathies make prompt diagnosis a challenge. Clinicians should be alerted and include amyloidosis in their differential diagnosis, particularly in patients with heart failure with preserved ejection fraction, unexplained left ventricle hypertrophy, particularly in those shown intolerance to previous antihypertensive medication, and early orthopedic manifestations of the disease such as carpal tunnel syndrome and spinal stenosis. The workup requires the exclusion of monoclonal gammopathies and technetium-99m pyrophosphate nuclear scintigraphy (^99m^Tc-PYP) studies with single-photon emission computerized tomography (SPECT) imaging. Several therapies are now available and can prolong life with significantly improved quality of life, particularly when the diagnosis of amyloidosis is made early. We present the case of a 77-year-old with a delayed diagnosis by five years to highlight the need for heightened clinical suspicion and an appropriate diagnostic algorithm for cardiac amyloidosis.

## Introduction

Cardiac amyloidosis is an infiltrative disease characterized by extracellular deposition of misfolded protein in the myocardium, leading to increased stiffness and eventual heart failure due to restrictive cardiomyopathy. Though there are more than 30 known amyloid types affecting humans, transthyretin (ATTR) and light-chain (AL) amyloidosis are the most likely to involve the heart [1].

AL amyloidosis is driven by a clonal gammopathy in the bone marrow, producing excess light chains. The incidence of the disease is estimated at approximately 3,000 cases/year in the US, with 50% of cases involving the heart. Disease-modifying treatment is guided by hematology-oncology providers.

ATTR amyloidosis results from dissociation, protein misfolding, and the deposition of beta-pleated sheets in end organs. Transthyretin, a tetrameric transport protein produced mainly by the liver, functions as a carrier for thyroxine and holo-retinol binding protein and travels in plasma as a tetramer [2]. When the tetramer dissociates into monomers, these aggregate into insoluble amyloid fibrils, which then deposit throughout the body. The stability of the tetramer can be degraded by genetic mutations leading to more rapid amyloid breakdown and accumulation, known as hereditary ATTR (hATTR). ATTR amyloidosis without genetic predisposition is called wild-type ATTR (wtATTR) and is more prevalent in elderly, predominantly male patients. Both are markedly underdiagnosed in the US. In an autopsy series of patients labeled with heart failure with preserved ejection fraction (HFpEF), 17% were found to have ATTR amyloid deposition [3]. Similarly, in an autopsy series of octogenarians dying for any reason, 25% were found to have myocardial amyloid deposition. Moreover, 5% of hypertrophic obstructive cardiomyopathy patients are thought to be misdiagnosed and have ATTR amyloidosis [1].

## Case presentation

A 77-year-old male with a history of hyperlipidemia, bilateral carpal tunnel syndrome (CTS), spinal stenosis (SS), and atrial fibrillation (AFib) was diagnosed with HFpEF in 2014, and shortly after, he came to our facility for consideration of AFib ablation. As part of his workup, he underwent cardiac magnetic resonance imaging (cMRI). He was found to have increased interventricular thickness and a dilated left atrium without reported increased interatrial thickness, and there was evidence of late gadolinium enhancement (LGE). The ablation was performed successfully, and no further workup was performed.

**Figure 1:**
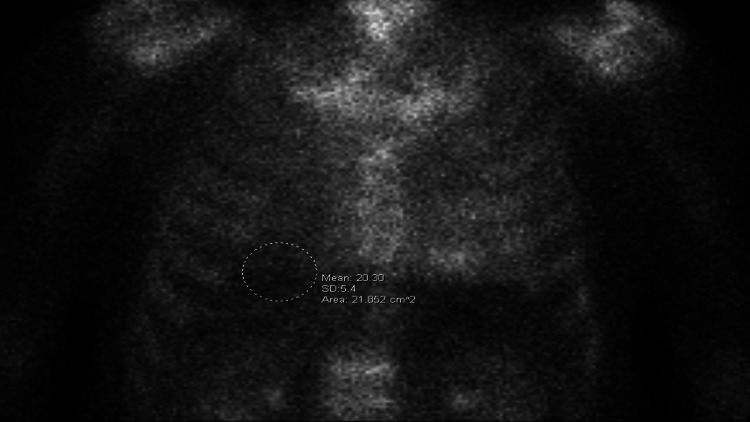
The patient’s technetium-99m pyrophosphate nuclear scintigraphy indicates a heart-to-contralateral lung ratio, calculated by comparing the tracer activity in the heart to that in the contralateral lung (dotted circle), of 1.6.

**Figure 2:**
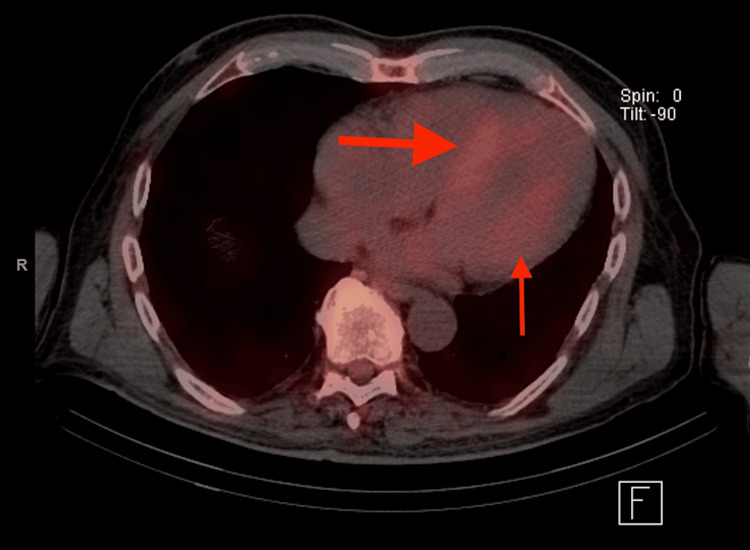
The patient’s technetium-99m pyrophosphate nuclear scintigraphy shows uptake limited to the myocardium (red arrows) on single-photon emission computerized tomography.

In 2019, five years after his abnormal cMRI, the patient was admitted with shortness of breath. His electrocardiogram (EKG) showed a recurrence of AFib with normal voltage. Troponin and brain natriuretic peptide (BNP) were elevated at 0.11 ng/dL and 259 pg/mL, respectively. An echocardiogram revealed left ventricular hypertrophy (LVH), an ejection fraction of 50%-55%, and biatrial dilation. He was cardioverted to sinus rhythm and discharged. During the post-discharge follow-up, cardiac biomarkers remained elevated. A repeat cMRI was ordered, which demonstrated an interventricular septum thickness of 1.5 cm (normal <1.1 cm), biatrial dilation, LGE in 75% of the subendocardial zones of the base, and relative sparing of the apex. These findings prompted an investigation into infiltrative cardiomyopathies. The serum kappa/lambda ratio was normal, and urine and serum protein electrophoresis (SPEP) with immunofixation electrophoresis (IFE) were negative for monoclonal gammopathy. A technetium-99m pyrophosphate nuclear scintigraphy (^99m^Tc-PYP) was ordered, revealing grade 3 uptake with a heart-to-contralateral lung ratio, calculated by comparing the tracer uptake in the heart to that in the contralateral lung, of 1.6 (Figure 1). The tracer uptake was limited to the myocardium on single-photon emission computerized tomography (SPECT) imaging (Figure 2).

Genetic testing was ordered, and upon returning negative, the patient was diagnosed with wtATTR.

Following the patient’s diagnosis, treatment with tafamidis was initiated for wtATTR, and a guideline-directed medical therapy was optimized for HFpEF, including loop diuretic therapy, spironolactone, and dapagliflozin. The patient continues to receive tafamidis treatment and has been tolerating it well for more than three years. After starting the treatment with tafamidis, loop diuretics, spironolactone, and dapagliflozin, he experienced a significant reduction in heart failure symptoms. There was a noticeable improvement in his quality of life and functional ability, as measured by the Kansas City Cardiomyopathy Questionnaire Summary Score (73-87 points) and the six-minute walk distance test (386-399 m), respectively. Notably, since starting the treatment, the patient has been hospitalized only once due to heart failure exacerbation. Furthermore, the patient was enrolled in a research study investigating the effect of a transthyretin gene-silencing medication in patients with wtATTR.

## Discussion

Common cardiac manifestations of ATTR amyloidosis include heart failure, restrictive cardiomyopathy, atrial arrhythmias, heart block, and aortic stenosis (particularly low-flow, low-gradient). EKG commonly reveals an inappropriately low voltage for the degree of LVH [1]. On echocardiograms, LVH, biatrial dilation, and an abnormal strain pattern with relative apical preservation are common. Laboratory results are frequently notable for stable, low-level elevation of troponin without another cause and high BNP levels. After ruling out the urgent diagnosis of AL, the diagnosis of ATTR can be confirmed by abnormal uptake on a ^99m^Tc-PYP study or by endomyocardial biopsy. The ^99m^Tc-PYP is highly sensitive (95%-99%) and specific (79%-100%) for ATTR cardiomyopathy. SPECT imaging is used to differentiate tracer uptake in the myocardium from the blood pool, a common cause of false-positive PYP scans performed without SPECT. Patients with abnormal AL laboratory workups or inconclusive non-invasive ATTR workups necessitate a biopsy-based tissue diagnosis.

**Figure 3:**
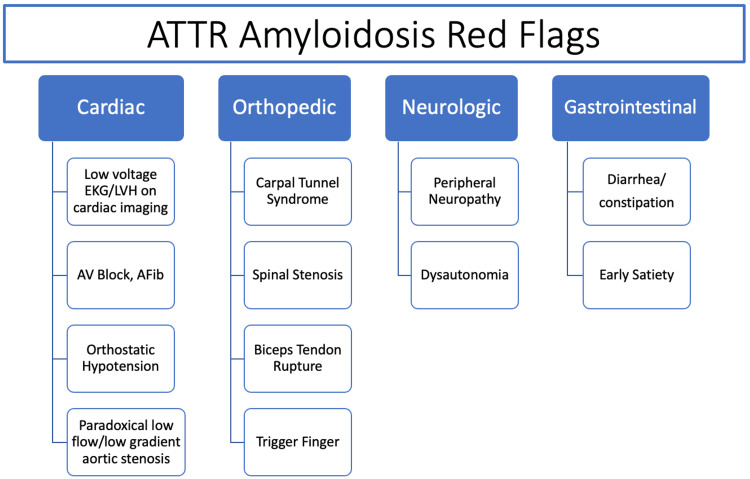
Flowchart of amyloidosis red flags. Image credits: Konstantinos Sideris. ATTR: transthyretin; LVH: left ventricular hypertrophy; EKG: electrocardiogram; AV: atrioventricular; AFib: atrial fibrillation.

Common non-cardiac manifestations of ATTR amyloidosis are driven by fibril deposition in the nervous system (polyneuropathy, orthostatic hypotension) and tenosynovium (CTS, SS, trigger fingers, ruptured biceps tendons). CTS is the most common initial symptom in over half of patients, preceding cardiac symptoms by a mean of 6.1 years and ATTR diagnosis by a mean of 6.9 years [4]. Symptomatic SS due to TTR ligamentum flavum deposition in both the cervical and lumbar regions is seen in up to 25% of ATTR patients [5]. Figure 3 shows a flow chart that summarizes clinical features that should be considered red flags and raise suspicion of amyloidosis.

Until recently, there were no treatments specific for ATTR amyloidosis. Several therapies are now available and can prolong life with significantly improved quality of life, particularly when the diagnosis of amyloidosis is made early. Transthyretin stabilizers, which include the approved tafamidis, as well as acoramidis, which is undergoing clinical studies, bind to the TTR protein and preventing its dissociation and subsequent misfolding. Transthyretin gene silencers, which include inotersen, patisiran, vutrisiran, and eplontersen, interfere with TTR mRNA and thus prevent the synthesis of the TTR protein. Transthyretin degraders are drugs under investigation that have the potential to selectively target deposited TTR proteins and lead to their removal from affected tissues through immune activation.

## Conclusions

Cardiac amyloidosis is an underdiagnosed condition affecting a larger portion of the population than was previously recognized. It is crucial for clinicians to be aware of the clinical red flags associated with amyloidosis to ensure a timely diagnosis. As currently available stabilizer and silencer treatments can only slow the progression of amyloidosis, it is imperative to diagnose ATTR and initiate therapy early before irreversible damage to the heart and other tissues occurs. Finally, new treatments are under investigation, offering hope for even better management of this condition in the future.
